# Whole genome sequencing for metastatic mutational burden in extraskeletal myxoid chondrosarcoma

**DOI:** 10.3389/fmmed.2023.1152550

**Published:** 2023-07-24

**Authors:** Trudy Zou, Rahil Sethi, Jiefei Wang, Gungor Budak, Uma Chandran, Ivy John, Rebecca Watters, Kurt Weiss

**Affiliations:** ^1^ Musculoskeletal Oncology Laboratory, Department of Orthopaedic Surgery, University of Pittsburgh School of Medicine, Pittsburgh, PA, United States; ^2^ Department of Biomedical Informatics, University of Pittsburgh, Pittsburgh, PA, United States; ^3^ Seven Bridges, Inc., Cambridge, MA, United States; ^4^ Department of Pathology, University of Pittsburgh School of Medicine, Pittsburgh, PA, United States

**Keywords:** sarcoma, soft tissue sarcoma, extraskeletal myxoid chondrosarcoma, structural variant, whole genome sequencing, lung metastasis

## Abstract

Extraskeletal myxoid chondrosarcoma (EMC) is an ultra-rare cancer that makes up less than 3% of all soft tissue sarcomas. It most often arises in the soft tissues of the proximal limbs and has a higher incidence in males. Though EMC has a good prognosis, it has an indolent course with high rates of local recurrence as well as metastasis to the lungs. EMC is characterized in 70% of cases by an EWS1-NR4A3 translocation, leading to constitutive expression of NR4A3. Structural variants (SVs) in EMC, especially large-scale genomic alterations, have not been well studied and studies are severely limited by sample size. In this study, we describe Whole Genome Sequencing (WGS) of a rare case of matched EMC primary tumor, lung metastasis, and pelvic metastasis to identify genomic alterations. We examined somatic variants, copy number variants (CNVs), and larger scale SVs such as translocations and breakend points. While the primary tumor and lung metastasis had similar somatic variations and CNVs, the pelvic metastasis had more unique SVs with especially increased mutational burden of SVs in chromosome 2. This suggests that different molecular drivers appear in more advanced, relapsing EMC compared with the primary tumor and early lung metastasis. Genomic studies such as ours may identify novel molecular complexities in rare cancers that may be leveraged for therapeutic strategies and precision medicine.

## 1 Introduction

Extraskeletal myxoid chondrosarcoma (EMC) is an ultra-rare soft tissue sarcoma with an estimated prevalence of 1/1,000,000 people (Goh et al., 2001). EMC most commonly arises in the deep tissues of the proximal extremities or limb girdles of males with a median age of onset of about 50 years. It has also been found in the head, neck, trunk, abdomen, retroperitoneal space, and bone. Though the name suggests it is of chondrocyte origin, EMC does not display a chondroid phenotype and is currently classified as a tumor of uncertain differentiation by the World Health Organization Classification of Tumors of Soft Tissue and Bone (WHO World Health et al., 2013).

EMC is in 70% of cases characterized by a t (9; 22) (q22; q12.2) translocation, fusing *EWSR1* to *NR4A3* and leading to constitutive expression of *NR4A3. NR4A3* can also fuse to TAF15 on chromosome 17 q12.2 in 20% of cases, which contributes a transactivation domain as well. Rarer *NR4A3* gene fusion partners occur in less than 5% of EMC cases. These include *FUS, TCF12,* and *TGF* (Hinrichs et al., 1985; Panagopoulos et al., 2002; Martínez-González et al., 2003; Broehm et al., 2014)*.* NR4A3 is a part of the NR4A protein family comprised of orphan nuclear hormone receptors. The mechanism by which this gene fusion leads to oncogenesis is unknown. Neural and neuroendocrine markers are frequently found in EMC, suggesting a possible neural/neuroendocrine differentiation in some cases (Harris et al., 2000; Oliveira et al., 2000; Goh et al., 2001; Okamoto et al., 2001).

EMC has an indolent course with a favorable prognosis compared with other soft tissue sarcomas: survival is approximately over 80% at 5 years and over 60% at 10 years (Drilon et al., 2008; Kapoor et al., 2014; Shao et al., 2016). However, it has high rates of local recurrence and metastasis to the lungs. Metastasis may also occur to bone, lymph nodes, soft tissues, and the brain. EMC is considered to be minimally responsive to chemotherapy, and standard treatment is surgical removal with wide margins. EMC in the metastatic stage is associated with much poorer prognosis due to limited systemic therapy options, with detection of metastases occurring from 6 to 15 years after initial diagnosis of the primary tumor (Meis-Kindblom et al., 1999). In one-third of cases, metastases are present before the primary tumor is detected or a definitive diagnosis is made (Fotiadis et al., 2005).

There is an unmet need to improve early detection of metastasis of EMC, as well as develop systemic therapy for recurrent disease. For ultra-rare tumors, understanding of molecular mechanisms that lead to oncogenesis and progression is severely limited by sample size and the rareness of these diseases. In this study, we examined rare, patient-matched samples of primary tumor (P), lung metastasis (LM), pelvic metastasis (PM), and peripheral blood monocytes (PBMC) in a patient with EMC. We conducted WGS to identify genomic variants from small nucleotide polymorphisms (SNPs) to SVs on the mega-base pair chromosomal scale. Identifying structural variants in rare tumors can yield understanding of molecular pathogenesis, staging of advanced disease, and potential therapeutic strategies for EMC.

## 2 Materials and methods

### 2.1 Tumor samples

**FIGURE 1:**
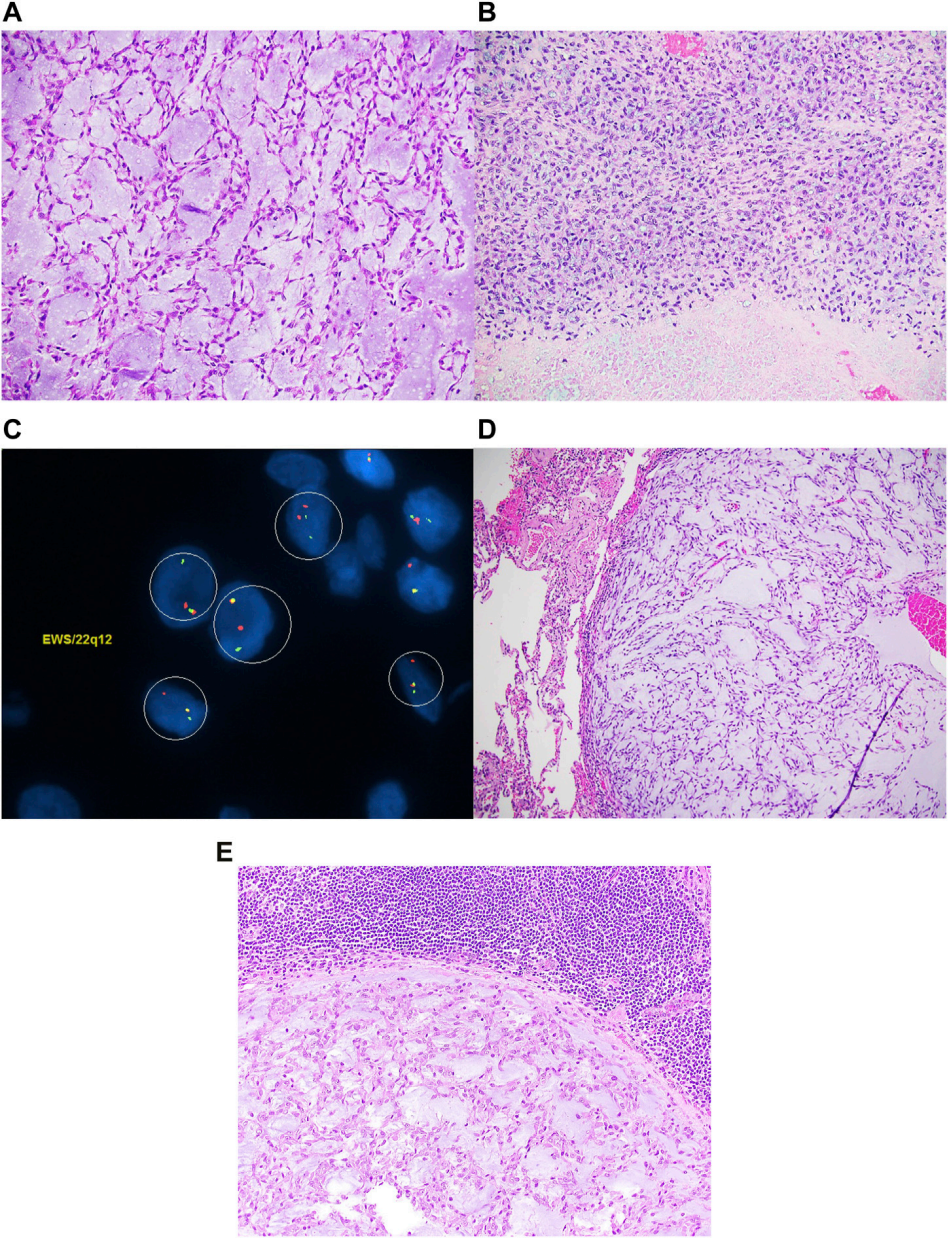
Extraskeletal myxoid chondrosarcoma composed of spindle cells arranged in a reticular architecture in a prominent myxoid stroma **(A)**. Focally, high-grade areas characterized by necrosis and sheets of large atypical epithelioid to spindle cells with coarse chromatin and prominent nucleoli are noted **(B)**. EWSR1 FISH studies are positive for gene rearrangement **(C)**. The tumor metastasized to the lungs **(D)** and lymph node **(E)**.

**FIGURE 2:**
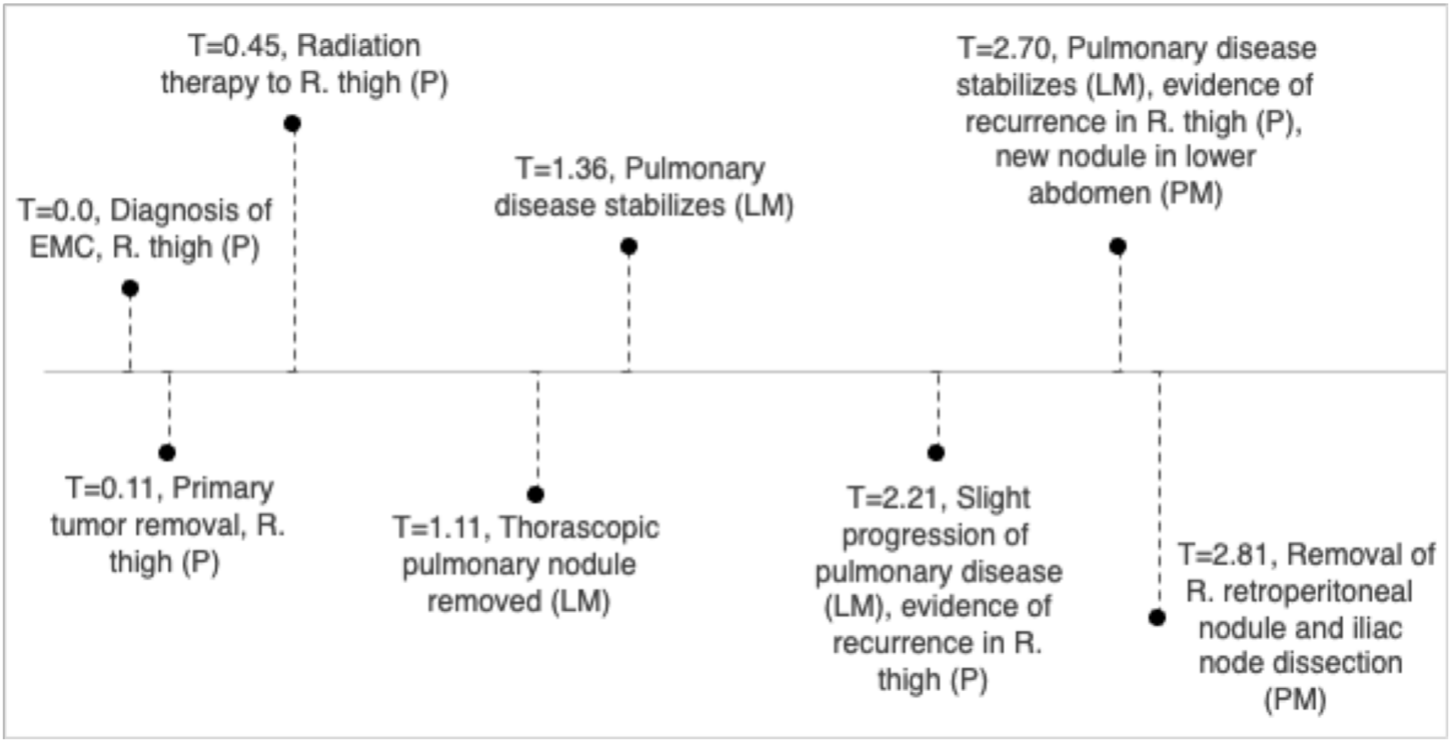
Clinical timeline, units in years with T = 1 indicating 1 year since diagnosis of EMC in right thigh.

An EMC was diagnosed histopathologically in a 57-year-old male with a primary tumor of his right thigh (Figure 1). Written, informed consent to participate in our Musculoskeletal Oncology Tumor Registry and Tissue Bank (IRB# STUDY20010034) was obtained. Sarcoma tissue was collected fresh from the operating room and processed immediately according to Pitt Biospecimen Core and Musculoskeletal Oncology Lab protocols. Subsequent metastases to the lung and pelvis were likewise collected fresh from the operating room and processed similarly. A summary of the patient’s clinical course is presented in Figure 2.

DNA was extracted from the frozen samples of the EMC P, LM, PM, and PBMC using the QIAamp Fast DNA Tissue Kit (Qiagen, Cat#51404). DNA concentration was determined with the Qubit 3.0 Fluorometer and DNA Broad Range Kit (ThermoFisher, Cat#Q32580). Samples were then submitted to the University of Pittsburgh Medical Center (UPMC) Genome Center (UGC) for further preparation, sequencing, and analysis.

### 2.2 Whole genome sequencing

The samples were processed following standard WGS pipeline in UPMC UGC. Extracted DNA was quantitated using Qubit™ dsDNA BR Assay Kit (Thermo Fisher Scientific). DNA libraries were prepared using the KAPA Hyper Plus Kit (Roche). 500 ng of genomic DNA was processed through fragmentation, enzymatic end-repair and A-tailing, ligation, followed by quality check using Fragment Analyzer (Agilent). The library size assessment was done using Fragment Analyzer (Agilent). Libraries with an average size of 400 bp (range: 200-600bp) were quantified by qPCR on the LightCycler 480 (Roche) using the KAPA qPCR quantification kit (Roche). The libraries were normalized and pooled by calculating the nM concentration based on the fragment size (base pairs) and the concentration (ng/mL) of the libraries by qPCR result. Sequencing was performed using NovaSeq 6,000 platform (Illumina) with 151 paired-end reads to an average target depth of 30–50X for germline and 87X, 74X, and 115X coverage for P, LM, and PM tumor samples respectively.

### 2.3 Somatic variant analysis

Concatenated base call files generated on NovaSeq 6,000 platform were converted to sample level FASTQ using Illumina’s bcltofastq v2.20. The quality of paired FASTQ reads was checked using FASTQC v0.11.3-1. The paired FASTQ reads were mapped to GRCh38 reference genome hs38DH (https://www.ncbi.nlm.nih.gov/pmc/articles/PMC5155401), duplicates were marked, and germline variants were called using Sentieon DNAseq pipeline v2.0.1-201911.012 (Kendig et al., 2019). Picard tools v2.4.1 (Broad Institute, 2016) were run on alignment BAM to provide mapping quality and metrics. The alignment BAM were processed with GATK and Mutect2 v4.0.5.1 (Benjamin et al., 2019) for somatic variant calling at tumor-normal mode as per GATK Best Practices (https://gatk.broadinstitute.org/hc/en-us/articles/360035894731-Somatic-short-variant-discovery-SNVs-Indels-) (Van der Auera, 2020), with PBMC sample as normal. The above pipeline was run on Linux-based AWS ec2 instances on DNAnexus^®^ platform. SAMTools v1.15.1 flagstat (Li et al., 2009) were run on processed BAM files to generate mapping stats. The called somatic variants were filtered on PASS filter. Filtered variants were annotated and converted from VCF to MAF format (https://docs.gdc.cancer.gov/Data/File_Formats/MAF_Format/) with vcf2maf.pl v1.6.21 (Kandoth et al., 2018) tool using VEP95 (McLaren et al., 2016) on GrCh38 annotation and hs38DH genome sequence. The MAF were further filtered for minimum total read depth (t_depth) of 25 and variant allele depth (t_alt_count) of 5. Using Maftools v2.8.5 (Mayakonda et al., 2018), Oncoplot and coOncoplot were generated from filtered MAF files, displaying somatic variants in protein coding genes.

### 2.4 Copy number variant analysis

CNV calling was performed on mapped BAM files from Sentieon DNASeq pipeline and somatic SNV VCF files from GATK Best Practices, using an in-house developed ensemble workflow. The ensemble workflow gathered and combined called results from CNV callers GATK cnv v4.0.5, CNVkit v0.9.5 (Talevich et al., 2016), CNVnator v0.2.7 (Abyzov et al., 2011), Manta v1.3.2 (Chen et al., 2016), and Sentieon CNV v201911 (Zheng et al., 2022) using SURVIVOR2 v1.0.3 (Jeffares et al., 2017). RandomForestClassifiers (trained with 1,000 Genome (1,000 Auton et al., 2015) samples) and event size were used for filtering. The filtered results were annotated with AnnotSV v1.1 (Geoffroy et al., 2018). For affected genes. CNVs with top 10 log2ratio values (deletion and duplication) from AnnotSV output and overlapping 1,000 base segments from GATK CNV calling pipeline were selected and converted to SEG files to view on IGV (Robinson et al., 2011).

### 2.5 Structural variant analysis

The alignment BAM files were uploaded to the Seven Bridges Cancer Genomics Cloud (CGC) (Lau et al., 2017). The Delly Somatic SV Calling app was used to run Delly 1.1.3 (Rausch et al., 2012) on all three tumor samples, each matched with PBMC control. The pipeline consists of somatic pre-filtering, re-genotyping of somatic sites, and post-filtering for somatic SVs. The BCFtools View app v1.15.1 was using to filter the results on PASS, compression level of 0, and INFO/IMPRECISE = 1. Telomeric and centromeric regions were excluded. The filtered results were annotated with AnnotSV v3.0.7 (Geoffroy et al., 2018). The filtered results were visualized using the CGC SVCircos app which is a wrapper around the circlize R package (Gu et al., 2014).

## 3 Results

### 3.1 Whole genome sequencing and mapping results

See Table 1 for mapping statistics generated using SAMTools. After filtering for PASS, the average raw depth of variant calling region was 59.52 for P, 52.66 for LM, and 58.11 for PM. After filtering reads for PASS, a tumor depth of 25, and an allele depth of 5, the remaining number of called variants were: 2,479 for P; 2,020 for LM; and 5,384 for PM. Of these, the number of called variants in the intergenic region (IGR), were: 1,663 for P; 1,296 for LM; and 3,142 for PM. P and LM shared 297 IGR variants, LM and PM shared 131 IGR variants, and P and PM shared 220 IGR variants. 259 IGR variants were shared by all three tumor samples. Of the non-IGR region variants, 202 were shared by P and LM; 54 were shared by LM and PM; and 122 were shared by P and PM. 128 were shared by all three samples.

**TABLE 1:** Mapping statistics generated using SAMTools v1.15.1 flagstat and depth.

	P	LM	PM	PBMC
Total (QC pass + QC failed)	893,578,786	760,459,060	1,176,896,493	557,693,299
#Primary reads	867,458,426	740,004,256	1,104,048,270	541,739,078
#Primary mapped reads	866,702,160	739,322,899	1,101,357,617	541,270,463
#Proper pair	844,104,934	719,133,284	1,015,786,012	525,108,642
%Primary mapped	99.91	99.91	99.76	99.91
%Proper pair	97.31	97.18	92.01	96.93
#Covered bases	2,718,402,310	2,718,022,683	2,720,851,858	2,716,363,876

### 3.2 Single nucleotide polymorphism in protein coding genes and mutation annotation

**FIGURE 3:**
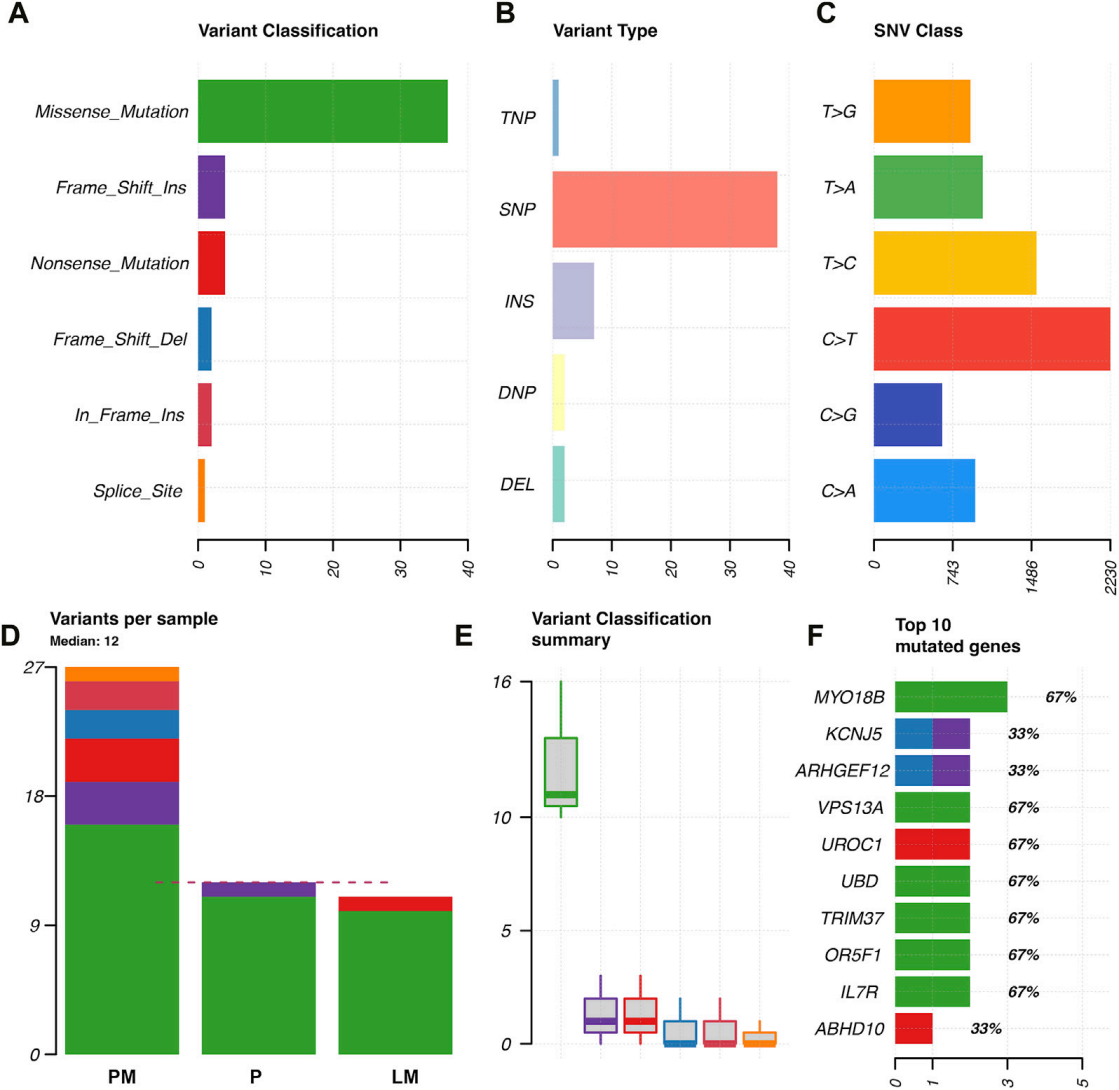
Characterization of variants found in GATK and mutect2 analysis of primary tumor, lung metastasis, and pelvic metastasis. **(A)** Variant classification. **(B)** Variant type (TNP = trinucleotide polymorphism, SNP = single nucleotide polymorphism, INS = insertion, DNP = dinucleotide polymorphism, DEL = deletion). **(C)** SNP classes. **(D)** Types of variants per sample, where dotted red line indicates median. **(E)** Variant classification summary showing range of frequency of variant types in samples. **(F)** Top 10 mutated genes in coding regions or splice donor/acceptor sites, x-axis = number of variants found, with variant type indicated by color. Numbers with percentages on the right of the bars represents percentage of samples in which gene was mutated (67% = mutated in two of three tumor samples).

**FIGURE 4:**
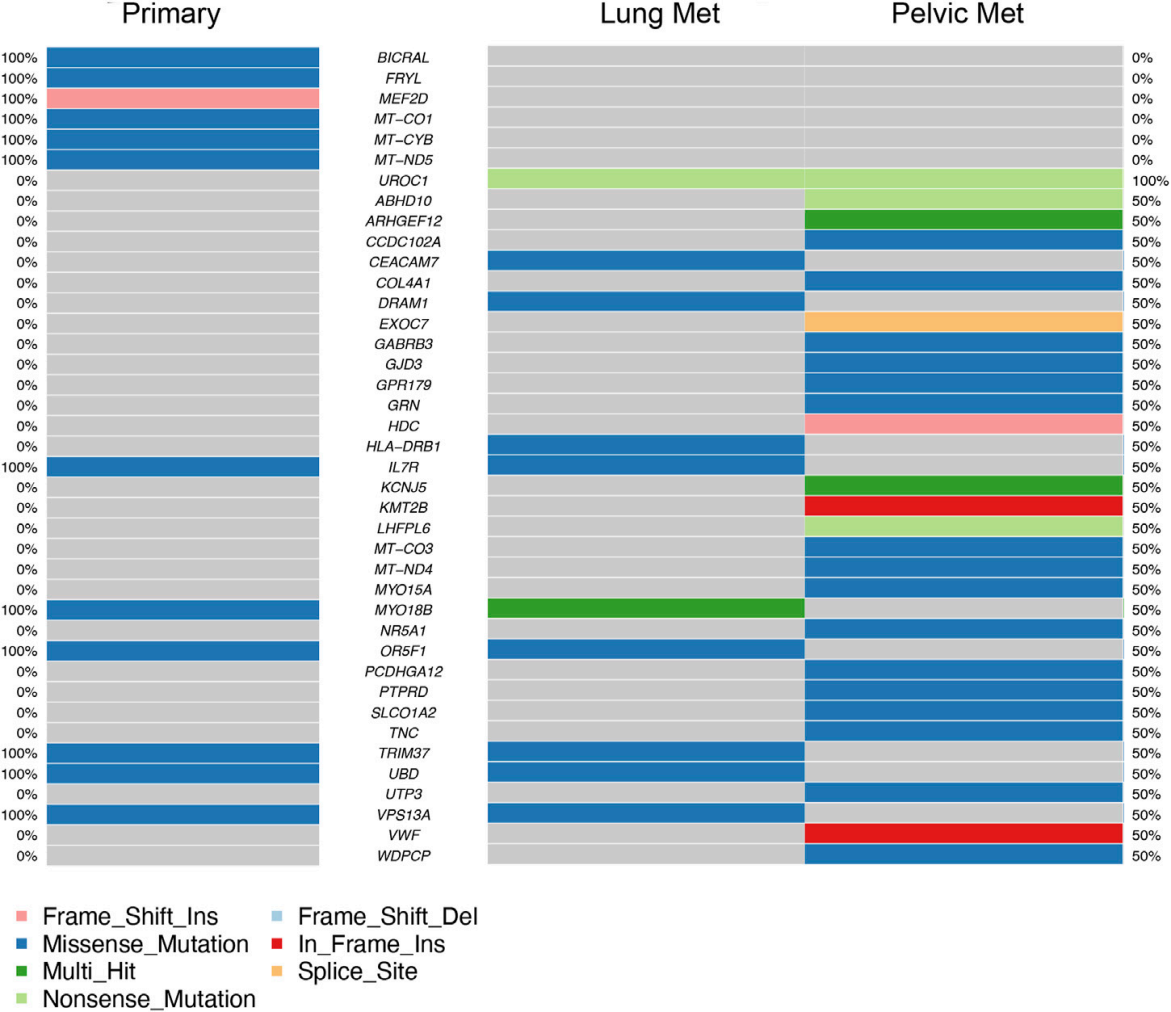
Top 40 genes with variants found in coding regions or splice donor/acceptor sites in primary tumor (left column) and metastases (right columns). Mutation type is represented by color in each gene. Percentages on far left and far right represent the percentage of samples the gene is mutated in, in primary tumor samples and metastatic samples respectively (ex. 50% = mutated in half of metastatic samples).

Coding region and splice/donor acceptor site variants identified by GATK and mutect2 in the three tumor samples were most frequently missense mutations; followed by frame shift insertions; then in-frame insertions, non-sense mutations, and frame shift deletions; and finally splice site mutations (Figure 3A). Variant types were most commonly SNPs, followed by insertions (Figure 3B). The most common single nucleotide variants found were mutations from C to T, while the reverse was the second most common single nucleotide variant found (Figure 3C). PM had the most variants overall (27 variants), followed by P (12 variants), and then LM (11 variants) (Figure 3D). Missense mutations had a large range of variants found in each sample compared to other variant types (16 in P, 11 in P, 10 in LM) (Figure 3E). *MYO18B* was the most commonly mutated gene, with one missense mutation found in P and two missense mutations found in LM (Figure 3F). One of these missense mutations, from A to T, was preserved from P (Supplementary Table S1). Overall, there were 40 genes total that contained at least one variant (Figure 4). P had 6 unique variants, LM had 3 unique variants, and PM had 24 unique variants not found in other samples (Figure 4). There were five variants, all missense mutations, that were shared by P and LM. These were in the genes *VPS13A*, *UBD*, *TRIM37*, *OR5F1*, and *IL7R* (Figures 3F, 4). One variant was shared between LM and PM: a non-sense mutation in *UROC1* (Figure 4). There were two genes that had both a frameshift insertion and a frameshift deletion in PM: *KCNJ5* and *ARHGEF12* (Figure 3F). There were no variants shared between all three tumor samples, nor were there variants shared by P and PM (Figure 4).

### 3.3 Copy number variant analysis

**FIGURE 5:**
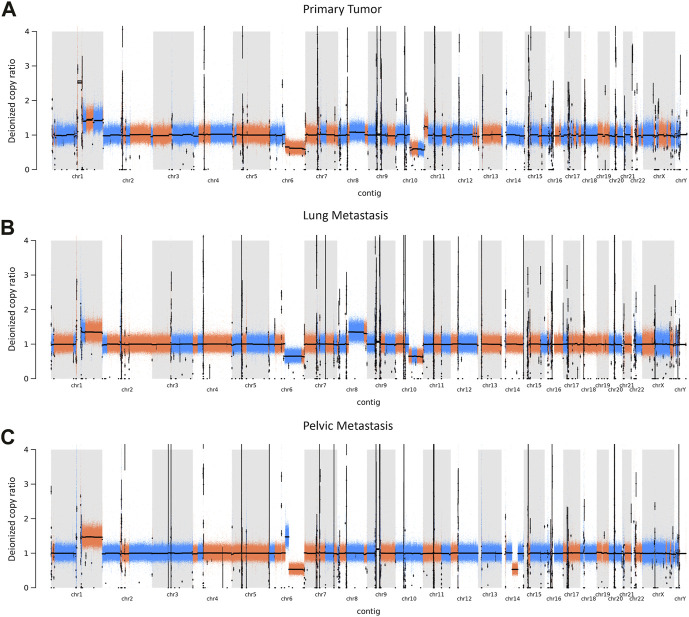
CNVs in EMC **(A)** primary tumor, **(B)** lung metastasis, and **(C)** pelvic metastasis. Colors represent a continuous copy number segment (alternate colors for each new segment), with black line being the average of the copy numbers of that segment (deionized copy ratio). Chromosome number is shown on the x-axis. CNV increase is represented by a deionized copy ratio > 1; while decrease is represented by deionized copy ratio < 1.

**FIGURE 6:**
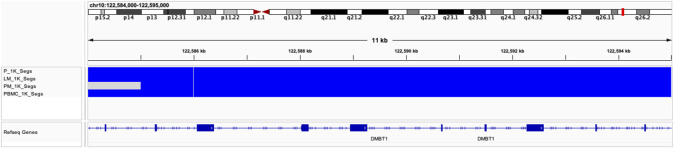
Copy number decreases found in DMBT1 shown in IGV browser. Blue indicates copy number decrease. Samples are indicated by row. From top to bottom: P, LM, PM, PBMC.

Global copy number increases shared between all three tumor samples included a copy number gain in chromosome 1q and a copy number loss in chromosome 6q (Figure 5). P and LM also shared a copy number loss in chromosome 10q (Figures 5A, B). LM had a unique copy number gain of chromosome 8q (Figure 5B), while the PM had a unique copy number loss in chromosome 14 (Figure 5C). At the gene level, *DMBT1* was found to have copy number decreases in all three tumor samples (copy ratio = 1.33E-09, 1.29E-09, and 4.14E-07 in P, LM, and PM respectively), as well as in PBMC (Figure 6; Table 2; Supplementary Table S2).

**TABLE 2:** CNV predicted segments found in *DMBT1* in all three tumor samples.

Sample	Gene	Caller	SV_start	SV_end	SV_type	CpRat
P	DMBT1	GATK	122584000	122595000	DEL	1.33E-09
LM	DMBT1	GATK	122584000	122595000	DEL	1.29E-09
LM	DMBT1	Sentieon	122585500	122593000	DEL	1.29E-09
PM	DMBT1	GATK	122586000	122595000	DEL	4.14E-07
PM	DMBT1	Sentieon	122585500	122592000	DEL	0.00028627

### 3.4 Structural variant analysis

**FIGURE 7:**
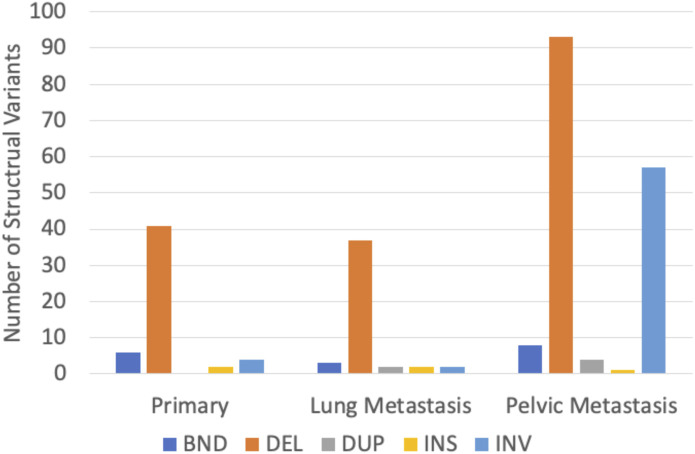
Distribution of structural variant types found in three EMC tumor samples. BND = break-end mutation, DEL = deletion, DUP = duplication, INS = insertion, and INV = inversion.

**FIGURE 8:**
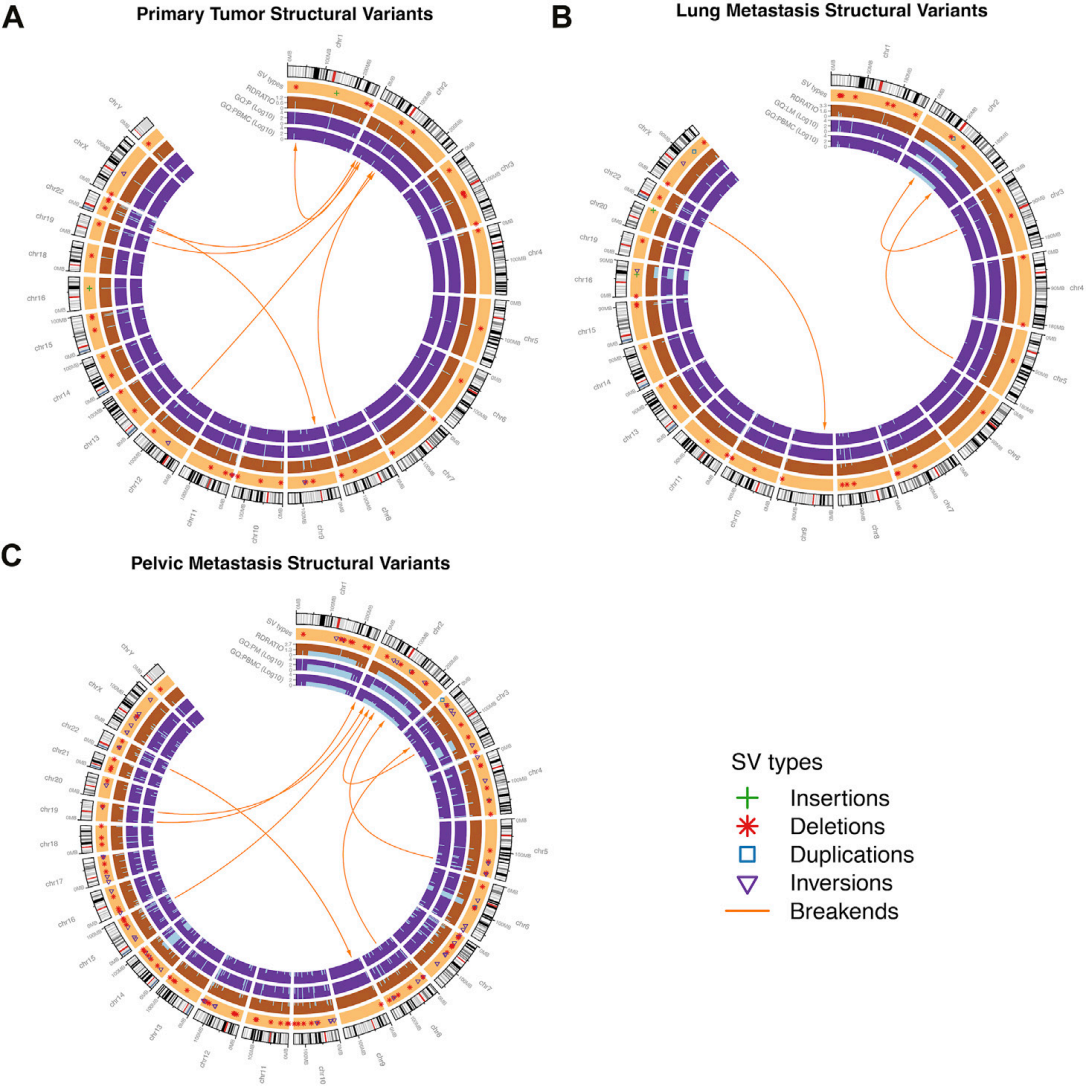
Structural variants (SVs) found in the in EMC **(A)** primary tumor, **(B)** lung metastasis, and **(C)** pelvic metastasis. Outer track segments represent chromosomes (black = heterochromatic region, white = euchromatic region, red = centromere). Specific SV types are shown in the light orange track. The dark orange and purple tracks represent read depth ratio (RD Ratio) and genotype quality (GQ) respectively, with GQ displayed for each sample (P = primary, LM = lung met, PM = pelvic met). Central orange arrows represent break-ends or inter-chromosomal translocations. Intra-chromosomal translocations are included in deletion, duplication, and inversion type SVs.

In P, 53 SVs were found, ranging from 26 bp to 206,360 bp, with a median length of 81 bp. In LM, 46 SVs were found, ranging from 38 bp to 121,506,824 bp with a median length of 84 bp. In the PM, 163 SVs were found, ranging from 28 bp to 184,523,626 bp with a median length of 98 bp. Deletions were the most common type of structural variant in all three samples (Figure 7). Inversions were significantly more frequent in PM compared to the other two samples (Figure 7). The characteristic t (9; 22) (q22; q12.2) translocation was identified by Delly, fusing EWSR1 to NR4A3, in all three tumor samples. This is indicated by a breakend (indicated by ‘BND’) SV on exon 12 of chromosome 22 to a coding sequence on chromosome 9, and *EWSR1* gene annotation (Supplementary Table S3). All samples contained frequent breakend mutations to chromosome 2 (Figure 8). Out of the breakend mutations that were not the characteristic EMC t (9; 22) translocation, 80% (4 out of 5) were to chromosome 2 in P, 100% (2 out of 2) were to chromosome 2 in LM, and 83% (5 out of 6) were to chromosome 2 in PM. One breakend SV, leading to an L1PA6 (member of LINE1 transposon family) insertion from chromosome 5q into chromosome 2q, was found in both LM and PM, but not P.

## 4 Discussion

EMC is an ultra-rare soft tissue sarcoma with an estimated prevalence of 1/1,000,000 people. Though indolent with favorable survival rates compared with other soft tissue sarcomas, EMC often demonstrates local recurrence and/or metastasis to the lungs and other sites. Detection of primary tumor or initial diagnosis before the onset of metastatic disease is essential for patients with EMC. Currently, no good prognostic biomarker exists for the detection of metastatic disease or prediction of aggressiveness of metastasis in EMC. Our results identified 1) somatic mutations in *MYO18B*, *TRIM37*, and *IL7R* as possible targetable drivers of oncogenesis, 2) a loss in copy number of chromosome 10q in primary tumor and lung metastasis samples, and 3) a high frequency of large-scale deletions and breakend point integrations into chromosome 2.

Somatic variant analysis identified six genes with mutations in coding regions in both P and LM: *MYO18B*, *VPS13A*, *UBD*, *TRIM37*, *OR5F1*, and *IL7R* (Figures 3F, 4). MYO18B has been found to be inactivated in about 50% of lung cancers by deletions, mutations, and methylation (Yokota et al., 2003). Restoration of MYO18B was found to suppress anchorage-independent growth in lung cancer, indicating a possible role for metastatic suppression for MYO18B in human lung cancer (Yokota et al., 2003). MYO18B was also found to be somatically mutated in poorly differentiated neuroendocrine carcinoma (Bhatla et al., 2016) and a possible tumor suppressor in medulloblastoma (Schieffer et al., 2019). MYO18B expression was not evaluated in our tumor samples, so the effects of the mutations found in our tumor samples should be investigated for a possible tumor suppressor role in EMC. *TRIM37* and *IL7R* both had missense mutations in primary tumor and lung metastasis and represent possible targets for gene therapy and mediation of chemoresistance. TRIM37 has been shown to promote chemoresistance and metastasis in triple-negative breast cancer (Przanowski et al., 2020). TRIM37 is linked to a 17q gain in neuroblastoma and 17q34 amplification in breast cancer (Meitinger et al., 2020), and has also been shown to cause genomic instability by delaying centrosome maturation (Yeow et al., 2020). Centrioles duplicate once a cell cycle in a process controlled by PLK4 (polo-family kinase). TRIM37 is a centrosomal ubiquitin ligase. When PLK4 is inhibited, low levels of TRIM37 can promote acentrosomal cell cycle proliferation, while high levels of TRIM37 inhibit acentrosomal proliferation. Thus, PLK4 inhibition is currently being explored in TRIM37-implicated cancers as a way to trigger selective mitotic failure for cancer treatment (Meitinger et al., 2020). Because EMC displays an unstable genome and is poorly responsive to chemotherapy, PLK4 inhibition may be explored as a possible therapy for EMCs that express high levels of TRIM37. The missense mutation found in our tumor samples should be investigated for its effects on TRIM37 expression. Finally, IL-7 and IL-7R play critical roles in the impaired immune systems of patients with cancer (Wang et al., 2022). IL-7 promotes an anti-tumor response in glioma, melanoma, lymphoma, leukemia, prostate cancer, and glioblastoma (Lin et al., 2017). In metastatic pediatric sarcomas including osteosarcoma and Ewing sarcoma, adjuvant therapy with recombinant IL-7 was shown to promote immune recovery and CD4 count recovery compared with standard therapy (Merchant et al., 2016). The immune profile and immunotherapy in EMC have been scarcely explored thus far. One recent trial of an antiangiogenic drug pazopanib in patients with advanced EMC identified lymphoid and myeloid infiltration in peri-tumoral areas and with tumor-associated macrophages intratumorally (Brenca et al., 2019). Currently, there is a Phase 1 trial recruiting patients with relapsed osteosarcoma and Ewing sarcoma for a trial of treatment with C7R (functionally active CD34-IL-7R) CAR-T cells (NCT03635632). Mutations in IL-7R in our primary tumor and lung metastasis may represent impaired IL-7 signaling in the tumor immune response. Immunotherapy should be further explored as a treatment, especially in metastatic relapsing EMC that requires systemic treatment. Overall, PM had the highest number of unique variants not found in the other two samples (24 unique variants). Since the PM sample had a higher number of reads mapped, no comparison can be made for the number of mutations found in each sample. The increased number of mutations in PM is likely due to a higher horizontal coverage (about 2 million more covered bases) (Table 1), as the tumor depth is similar for all samples (around 50). However, the results do show the variants implicated in P and LM are different than the variants implicated in PM. Thus, different somatic tumor drivers likely appear with more advanced disease.

Our data shows copy number gains in chromosome 1q and copy number losses in chromosome 6q in all three samples (Figure 5). These have previously also been identified in EMC by Davis et al. (2017). In particular, the Davis et al. study identifies copy number gains in chromosome 1q in four out of six EMC cases, with three of these being metastatic lung samples and one being a metastatic gluteus sample. The Davis et al. metastatic gluteus sample shows many CNV mutational similarities to our LM sample at the chromosome level, also sharing a copy number decrease in chromosome 6q and copy number increase in chromosome 8q. These CNVs found in our study highlight the importance of increased data and sample size for ultra-rare cancers such as EMC. The Davis et al. study additionally identified somatic point mutations that they reported were of ‘unknown significance’. Our data did not share any somatic point mutations with their samples, supporting their hypothesis that the mutational profile of metastatic EMC is limited beyond the pathognomonic translocation, at least with respect to somatic point mutations. In our study, P and LM also shared a copy number decrease in chromosome 10q (Figures 5A, B). Partial or entire losses of chromosome 10q is frequent in a large variety of human cancers, including glioblastomas, carcinomas, lymphomas, and melanomas (Fujisawa et al., 2000). Many tumor suppressor genes have been identified on chromosome 10q, including *PTEN* at 10q23.3, *LGI1* at 10q24, *BUB3* at 10q24-q26, *MX1* at 10q25.1, *LIMAB1* at 10q25, and *DMBT1* at 10q26.1 (Fujisawa et al., 2000). *DMBT1* showed the most significant copy number decrease of any gene that had copy number decreases in all three EMC tumor samples in our data (copy ratio = 1.33E-09, 1.29E-09, and 4.14E-07 in primary tumor, lung metastasis, and pelvic metastasis respectively). *DMBT1* (deleted in malignant brain tumors 1) codes for a glycoprotein containing multiple scavenger receptor cysteine-rich (SRCR) domains. It is often deleted in glioblastoma (Mollenhauer et al., 1997) and is considered a candidate tumor suppressor gene in brain, gastrointestinal, and lung cancer; as well as having a role in immune defense (Mollenhauer et al., 2000). In glioblastoma, chromosome 10 is entirely lost in primary glioblastomas, while only chromosome 10q is lost in secondary glioblastoma, suggesting the involvement of different tumor suppressor genes (Fujisawa et al., 2000). It suggests there may be a severe loss of tumor suppressor genes on chromosome 10q in P and LM, including *DMBT1*. In summary, all three tumors share copy number gains in chromosome 1q and losses in chromosome 6q, while P and LM share a copy number loss in chromosome 10q. The significance of these CNVs in EMC biology should be further explored.

Delly SV Analysis was able to identify the characteristic t (9; 22) translocation unidentified by other methods of genomic analysis in all three samples. The identification of the translocation in all three samples demonstrates that this translocation is a conserved mutation that is likely important to EMC biology. Additionally, it supports Delly as a reliable tool for SV detection. P and LM were more similar in their SV mutational burden, while more SVs were identified in the PM as well as longer SVs. This is again likely due to the significantly larger number of mapped reads for PM. However, the frequency of inversions also greatly increased in PM, indicating increased genome instability. Deletions were the most common type of SV found by far. Taking the SNP and CNV findings into account, this could indicate that pathogenesis in EMC is generally driven by accumulation of loss of tumor suppressor genes. Interestingly, all tumor samples shared high rates of breakend mutations to chromosome 2. LM and PM shared a breakend mutation from chromosome 5q to chromosome 2q. This breakend SV was annotated for L1PA6, a member of the LINE1 (long interspersed nuclear element-1) transposon family. LINE1 transposons comprise approximately 17% of the human genome, and somatic LINE1 integrations are present in almost half of all cancers. The known role of LINE1 in cancer in the currently literature is primarily through epigenetic regulation and hypomethylation of LINE1 (Zhang et al., 2020). Aberrant LINE1 integrations also play a role in oncogenesis; aberrant integration leads to megabase scale chromosomal rearrangements that can result in removal of tumor suppressor genes or trigger the amplification of oncogenes (Rodriguez-Martin et al., 2020). PM contained another breakend mutation from chromosome 3p to chromosome 2p, encoding L1PA3, another LINE1 element. In all samples, the majority of breakend mutations result in chromosome 2 integrations. To our knowledge, large-scale structural variants of insertions, inversions, and breakends have not been studied in EMC thus far. Our data reveal a possible role of chromosome 2 in the progression of genome instability of EMC, and increased incidence of LINE1 insertions with tumor progression. The effect of LINE1 insertions and the significance of chromosome 2 insertions should be further studied to determine if there is an oncogenic role in EMC beyond general genome instability.

Our study has several limitations. First, our sample size was limited to one case of matched primary tumor and two metastatic tissues in the same patient at different time points in his clinical course. This limits the generalizability of our data to other cases of EMC. Secondly, while WGS allowed us to identify mega base-scale chromosomal somatic mutations including translocations, our findings must be verified with transcriptomics and RNA/protein expression. Somatic variant analysis in this study focused on protein-coding genes, with the goal of identifying oncogenic drivers or therapeutic targets, but should be expanded to non-coding regions and non-intergenic regions in future studies. Finally, in CNV analysis, CNVs for PBMC were separately generated and then compared with tumor samples, instead of PBMC being used as the matched normal as with somatic and SV analysis. A pooled normal analysis decreases noise and resulted in better CNV analysis. However, this also limited our identification of somatic CNVs, such as *DMBT1* copy number decrease, indicating that these findings may represent germline mutations. It is also possible that germline mutations contributed to the overall genome instability in our EMC samples. *DMBT1* germline deletions have been found in neuroendocrine tumors (Qu et al., 2020) hemizygous germline deletions of *DMBT1* have been found in primary brain tumors (Pang et al., 2003).

Our overall results in somatic mutation, CNV, and SV show more similarities between the primary tumor and the lung metastasis than with the pelvic metastasis. This is likely due to timeline of metastatic disease. In our patient, the pulmonary nodule was removed 1.11 years after initial diagnosis, with pulmonary disease stabilization at 1.36 years. However, at 2.7 years, there is evidence of both recurrence in right thigh and the appearance of the pelvic metastasis in an iliac lymph node. Thus, the pelvic metastasis may have metastasized from new mutational events that also resulted in local recurrence. The initial EMC may have been driven by SNPs in *MYO18B*, *TRIM37*, and *IL7R*; or loss of tumor suppressor genes on chromosome 10q. The more aggressive pelvic metastasis appears with unique SNPs, and a higher mutational burden of larger size structural variants such as inversions. There may be hypermutation due to the increased number of LINE1 integrations or other random events, especially in chromosome 2, as the disease progresses.

In conclusion, our data suggest that there may be unique genomic mutations in more advanced and recurrent EMC. Further studies will focus on tumor phylogenetics to identify if the pelvic metastasis was an independent clonal evolution from the primary tumor, as opposed to from the lung metastasis, and the mechanisms underlying its more unique phenotype. These alterations should be further studied to improve outcomes for patients with advanced EMC.

## Data Availability

The original contributions presented in the study are publicly available. This data can be found here: [Link: https://www.ncbi.nlm.nih.gov/projects/gap/cgi-bin/study.cgi?study_id=phs003305.v1.p1/ Accession numbers: STUDY: phs003305; SAMPLE: S4 (SAMN35440161); EXPERIMENT: S4 (SRX20750599); RUN: PM_WAT403A2_S11_001_markdup_realigned.bam (SRR24994636); SAMPLE: S3 (SAMN35440162); EXPERIMENT: S3 (SRX20750600); RUN: LM_S16_markdup_realigned.bam (SRR24994637); SAMPLE: S1 (SAMN35440160); EXPERIMENT: S1 (SRX20750602); RUN: PBMC_S18_markdup_realigned.bam (SRR24994639); SAMPLE: S2 (SAMN35440159); EXPERIMENT: S2 (SRX20750601); RUN: P_S15_markdup_realigned.bam (SRR24994638)].
